# Microbial dysbiosis in melasma through community profiling

**DOI:** 10.3389/frmbi.2025.1505565

**Published:** 2025-12-22

**Authors:** Yugandhar Reddy B.S, Chandraprabha Doraiswamy, Deepshikha Singh, Nagalakshmi Surendra, Aparna Damle, Maitreyee Dutta, Savitha Rajkumar, Brian Potterf, Arindam Roy, Bharat Cheviti, Tony Dadd, David Arnold, Sarah Paterson, Mukta Sachdev, Paul Van-Der Logt, Nirmala Nair, Nagasuma Chandra

**Affiliations:** 1 Unilever Research & Development, Bangalore, India; 2 Department of Biochemistry, Indian Institute of Science, Bangalore, India; 3 Unilever Research & Development, Trumbull, CT, United States; 4 Unilever Research & Development, Colworth Science Park, Bedford, United Kingdom; 5 Unilever Research & Development, Port Sunlight Research Lab, Birkenhead, United Kingdom; 6 MS Clinical Research, Bangalore, India; 7 Unilever Research & Development, Wageningen, Netherlands

**Keywords:** skin microbiome, melasma, community network analysis, microbial dysbiosis, clinical analysis

## Abstract

**Background:**

The complex ecosystem on skin comprising tens of thousands of microorganisms plays an important role in health and disease. The last decade in particular has witnessed a surge in microbiome research, which has been elucidating the role of the microbiota in numerous skin pathologies. Of relevance to the current study, are recent evidences implicating the microbiome in skin pigmentary conditions. Melasma is one such refractory, hyperpigmentary condition with a poorly understood pathogenesis. The present study was carried out to characterize the nature of microbial dysbiosis and its impact on microbial community structure in melasma subjects.

**Results:**

The clinical assessment of melasma carried out using biophysical, biochemical and biomarker-based measures confirmed significant changes in melasma lesions, most notably, those linked to redox, inflammation and barrier properties. A deep characterization of the skin microbiome in melasma from human face, indicated significant differences between lesional and peri-lesional areas. Of the 377 genera identified through an agglomeration of all OTUs at the Genus level through 16S rRNA sequencing, 344 were common, while 12 were unique to lesional and 21 unique in peri-lesional areas. A significant decrease was observed in alpha diversity in melasma lesion as compared to peri-lesion areas, with an accompanying decrease in number of interconnections among them. The differences in the microbiome also appeared to correlate with several clinical parameters, notably with the melasma severity measured through modified MASI (mMASI) scoring. The observed changes in both host and microbiome, point to a potential role for the latter in melasma pathogenesis.

**Conclusions:**

Our study indicates that there are significant differences in the microbiome between lesional and peri-lesional areas of melasma subjects, with associated changes in microbial community structures. Additionally, the observed changes were seen to correlate with measured clinical parameters. These findings provide the opportunity to further probe the nature of host and microbiome links that may underlie the phenotypic manifestation, as well as provide effective routes for managing this recalcitrant disorder.

## Introduction

Skin, the largest organ in the human body harbours millions of bacteria, fungi and viruses that comprise the skin microbiota, most of them being beneficial and non-pathogenic in nature (Grice and Segre, 2011). The microbiota participates in protecting against invading pathogens, participating in immune responses, facilitating nutrient availability, etc., while the host in turn provides nutrients and a stable ecosystem. Such mutually beneficial interactions are mediated by molecular interactions between microbes and host molecules such as proteins, RNA and metabolites (Sudhakar et al, 2021).

In the last decade, advances in DNA sequencing and culture-independent techniques that employ sequencing of marker genes, such as the bacterial-specific 16S ribosomal RNA (rRNA) gene, coupled with unique *in vitro* model systems and new methodologies for high-throughput profiling of multiple -omics data types have accelerated the pace of microbiome research and provided enormous information on community characteristics of microorganisms. From a historical perspective, early skin-based studies carried out using bacterial 16S ribosomal RNA (rRNA) gene amplicon-based sequencing methods revealed topography-dependent differences in microbial communities that correlated with the three main skin microenvironments, namely moist, dry and sebaceous. Over time, the application of shotgun metagenomics, significantly improved our understanding of microbial communities, including multi-kingdom analysis (fungi and viruses, besides bacteria), species and strain-level understanding of microbiota and temporal stability of the microbiome (Bouslimani et al, 2015; Costello et al, 2009; Grice et al, 2009; Human Microbiome Project Consortium, 2012; Dorrestein et al, 2018; Flowers and Grice, 2020). These technological advances also encouraged investigations into the nature of host-microbe interactions, particularly, because perturbations in the balance between commensals and pathogens and a breach in the skin barrier were shown to be increasingly associated with skin diseases (Schommer and Gallo, 2013; Byrd et al, 2018). Notable skin conditions for which discriminatory taxa and microbiome signatures have been deciphered include atopic dermatitis (Geoghegan et al, 2018; Paller et al, 2019; Bjerre et al, 2021), acne (Barnard et al, 2016; Kim et al, 2021); psoriasis (Tett et al, 2017; Langan et al, 2019) and dandruff (Grimshaw et al, 2019; Wang et al, 2022). Despite these advances, what is still poorly understood is how the microbial dysbiosis is causally linked to these phenotypes. While this has been unequivocally established in a few cases, notably atopic dermatitis (Fyhrquist et al, 2019) and diabetes (Qin et al, 2012; Zhao et al, 2023), in several others, it is still not. Deciphering such linkages of the microbiome to disease pathology is likely to contribute novel therapeutic strategies to ameliorate disease states.

Of interest to the current study are recent evidences pointing to microbial dysbiosis in skin pigmentary conditions (both hypo- and hyperpigmentation). On the one hand, studies have reported microbial dysbiosis in diseases such as vitiligo, characterized by patchy, white skin owing to melanocyte loss (Gangu et al, 2016) and links between skin dysbiosis, mitochondrial damage, and immunity in patients with vitiligo (Bzioueche et al, 2021). On the other hand (Zanchetta et al, 2022), reported specific microbiota composition and networks on skin based on hyperpigmentation status, through their study on solar and senile lentigo. Melasma, an acquired disorder of hyperpigmentation (sometimes referred to as chloasma or the mask of pregnancy), presents as light brown to dark brown patches with irregular and symmetrical borders. Sun exposed facial areas such as the cheeks, forehead, and upper lip are commonly involved, with the central facial region being affected in 50–80% of cases. While it can occur in both sexes, women (accounting for 90% of all cases) and individuals with darker complexions (Fitzpatrick’s skin types IV to VI), especially those living in areas of intense UV radiation, such as Hispanics/Latinos, Asians, and African Americans are most afflicted. Epidemiological studies estimate the prevalence of melasma in the general population is at 1% and in higher-risk populations at 9–50%, though true prevalence across the entire population is unknown. Predisposing factors include pregnancy, genetics, sun exposure, endocrine dysfunction or hormone treatments, use of oral contraceptives, as well as cosmetics and drugs containing phototoxic agents (Gupta et al, 2006; Rendon et al, 2006; Handel et al, 2014; Ogbechie-Godec and Elbuluk, 2017). Current treatment options being used for the clinical management of melasma include a diverse array of topical preparations including hydroquinone and the triple combination, azelaic acid, niacinamide, vitamin C, kojic acid, and actives such as cysteamine and thiamidol, systemic treatments such as tranexamic acid and combinations with topical therapies, and lastly, superficial peels and laser therapy (Cassiano et al, 2022). Despite the numerous options, the recalcitrant and recurrent nature of melasma often leads to poor treatment outcomes and severely impairs quality of life of affected individuals, thus warranting the development of newer treatment strategies.

Interestingly, a study by Hu et al. pointed to skin microbiome dysbiosis in melasma (Hu et al, 2022). More recent studies have reported an association between the gut microbiota and melasma via the body’s oestrogen metabolism (Liu et al, 2022). However, focused studies investigating the specific nature of dysbiosis in melasma lesions and its links to the phenotype is lacking. With this background, we aimed to characterize the nature of the microbial dysbiosis and its impact on microbial community structure in 40 melasma subjects. We also queried potential linkages to the phenotype through a range of measured clinical parameters. The findings can deepen our understanding of the role of the microbiome in disease severity and/or progression and/or heterogeneity. Also, restoring the skin microbiome composition could be an effective and simple treatment strategy owing to the ease at which it can be administered.

## Materials and methods

### Ethics approval and consent to participate

The study protocol was approved by CLINICOM Independent Ethics Committee for Evaluation of Protocols for Clinical Research (ECR/64/Indt/KA/2013). All study participants gave their written informed consent before participating in the study.

### Consent for publication

Written informed consent given by study participants covered consent for publication.

### Study population

Participants who were willing to provide written consent were considered in the study. Forty otherwise healthy, female Indian participants, aged 25–45 years old, with Skin Type III to IV presenting with 50 to 69% (as per the area scores of the mMASI scale) coverage area of melasma on both sides of the cheeks and with moderate to severe facial melasma that was stable for 6 months were included in the study. Melasma severity was determined by dermatologists using the mMASI scale (Pandya et al, 2011). Pigment type (epidermal/dermal/mixed) was determined using the Woods Lamp (Katsambas and Antoniou, 1995). Medical history was obtained from every study participant. Details related to inclusion/exclusion criteria are presented in **Supplementary Data Sheet S1**. Briefly, participants who underwent any treatment for melasma, such as laser therapy, bleaching agents, or any other skin lightening treatment (topical or oral) in 6 months prior to sample collection, used oral contraceptives or on hormone replacement therapy, topical antibiotics or topical steroids past 4 weeks, took systemic antibiotics (oral, intravenous, intramuscular), oral corticosteroids, cytokines or immunosuppressive agents 6 months prior to sampling or participants who are pregnant, lactating or nursing, were excluded from the study. Representative images of the lesions are presented in **Supplementary Data Sheet S2**.

### Study outline

The study was conducted for a period of about 2 weeks for each subject and included a total of 4 visits i.e., Visit 1 (enrolment and screening visit), Visit 2 (assessment visit), Visit 3 (assessment visit) and Visit 4 (final visit). Details related to study visits are outlined in **Supplementary Data Sheet S3**.

### Microbial profiling by 16S rRNA method

#### Microbiome sampling

Participants were asked to refrain from using any products containing anti-microbial or antiseptics on their face throughout the study. Participants did not wash their face for at least 24 hours prior to the day of microbial sampling. They were acclimatized in a temperature and humidity-controlled (22°C+/-2°C and relative humidity of 50%+/- 10%) clean and dry room for at least 2hrs on the day of sampling.

Skin bacteria were collected from right and left melasma lesion areas and likewise from right and left peri-lesional areas on the face using non-invasive sterile swabs moistened with sterile Phosphate Buffered Saline (pH 7.4) containing 0.1% Triton X 100. Swabs were transferred to sterile tubes containing lysis buffer for subsequent DNA extraction. Each left cheek sample was pooled with respective right cheek sample (separately for lesional and peri-lesional swabs). Samples were stored at -20°C until further processing.

#### DNA extraction, library preparation and sequencing of 16s rDNA samples

DNA extractions were carried out using the QIAamp BiOstic Bacteremia kit (Qiagen, #12240-50). DNA was quantified using Qubit dsDNA HS Assay kit (ThermoFisher Scientific, #Q32854). 16S PCR was carried out as a part of the QC process using universal 16S primers (listed below) using the following thermal conditions: Initial denaturation at 95°C for 3mins, 35 cycles of – 95°C for 30sec, 55°C for 30sec and 72°C for 60sec – final elongation of 72°C for 7mins. The PCR product (~1.5kb) was then checked on 1% Agarose gel to confirm the presence of 16S rDNA.

16SF 5’-AGAGTTTGATCCTGGCTCAG-3’.

16SR 5’-GGTTACCTTGTTACGACTT-3’.

Amplification of V3-V4 region was carried out using bacterial specific primer pair (listed below) for each of the pooled sample using 16S product as the template in a nested PCR.

V3V4F 5’-CCTACGGGNGGCWGCAG-3’.

V3V4R 5’-GACTACHVGGGTATCTAATCC-3’.

The V3-V4 PCR products were processed for library preparations to make the DNA molecules compatible for sequencing on Illumina HiSeq platform. NEB Ultra DNA library preparation kit was used (NEB, #E7370) for this purpose. Briefly, the products were end-repaired and monoadenylated on the 3’ ends. This is followed by sticky end ligation of Illumina indexed adapters which are complementary to the lawn of oligos on the flow cells. The adapter ligated molecules were then enriched using specific primers with 10 cycle PCR. Next, the PCR products were cleaned using AMPure XP beads (Beckman Coulter, #A63880). The cleaned product was quantified using Qubit dsDNA HS Assay kit followed by fragment size distribution analysis on D1000 TapeStation Screen Tapes (Agilent Technologies, # 5067-5582).

The 16s rDNA samples (V3-V4 amplicons) were sequenced using Illumina HiSeq2500 platform with a pair end approach using 250 paired end chemistry. Data quality was assessed before proceeding with further analysis. The analysis pipeline is outlined in **Supplementary Data Sheet S4**.

### Microbiome community structure analysis

The community structure network analysis was carried out by removing taxa which were not present it at least 40% of the samples and those whose total counts were less than 10. Co-occurrence analysis was done using the SpiecEasi R package and networks were plotted using iGraph, ggraph and ggplot2 packages.

### Skin barrier measurements & biomarker analysis through tapestripping

Each subject washed their face with water and were acclimatized for approximately 15 to 30 mins at temperature 20-22°C and relative humidity at 40% - 60%. TEWL was recorded two hours post face wash at lesional and peri-lesional sites using a Tewameter (TM 300 Courage-Khazhaka). One of the sides of the face was selected for tape stripping using D-squames as per the randomization. TEWL was measured at various timepoints on the selected side at baseline, after 5^th^ and 10^th^ tape strips (D-squame–2.2 cm), 5 hours (+/- 30 mins) and 24 hours after the pre-tape stripping baseline measurement. Tape strips were stored at -80°C until further processing.

### Stratum corneum and biomarker analysis

Tapestrips were processed for stratum corneum properties and biomarkers influencing multiple skin functions. Towards this, the first tape strip was discarded and the remaining nine were used for subsequent measurements. Tapestrips were evaluated for a) barrier function through Natural Moisturizing Factors (NMFs) [free amino acids (FAAs) and glycerol (GLYC)] and cohesivity (stratum corneum total protein content), b) inflammatory status through evaluation of Interleukin-1α (IL-1α), IL1receptor antagonist (IL-1RA), IL-1RA/IL-1α ratio and c) redox mechanistic understanding, enzymatic antioxidants [Catalase (CAT), superoxide dismutase (SOD)] and total non-enzymatic anti-antioxidant activity (ToAx). Catalase, SOD, Total antioxidant activity, glycerol content, free amino acid content, and IL-1α and IL-1RA content were measured in each D-squame layer. Further details on methods adopted to evaluate stratum corneum properties and biomarkers is outlined in **Supplementary Data Sheet S5**.

### Statistical analyses

Statistical analyses were performed using SAS (Statistical Analysis System) v9.4 and base R. All Statistical analysis was carried out at significance level of α = 0.05. ​​Standard linear mixed modelling techniques were used to analyse each endpoint and estimates for differences between lesional and peri-lesional sites were produced as model output, together with p-values and 95% CIs (Confidence Interval). Where clinical parameters showed skewed distributions, they were first log-transformed, before carrying out the tests. Baseline measurements where available, were considered as covariates. Genera were filtered across all samples by a prevalence of 60%. Differences in the relative abundance of the prevalent genera were calculated for each pair of lesion and peri-lesion. Comparison between clusters & singleton were evaluated using ANOVA followed by the Tukey HSD test. Pairwise correlations using Pearson correlation across all sample pairs were calculated using the differences in relative abundance, and further used for unsupervised hierarchical clustering using base R. Correlation estimate (R) across pairs within a cluster was noted to determine the optimal tree height to determine the clusters. Data was represented as boxplot and correlogram using R.

## Results

### Melasma lesion versus peri-lesion areas are associated with altered microbiomes

**Figure 1:**
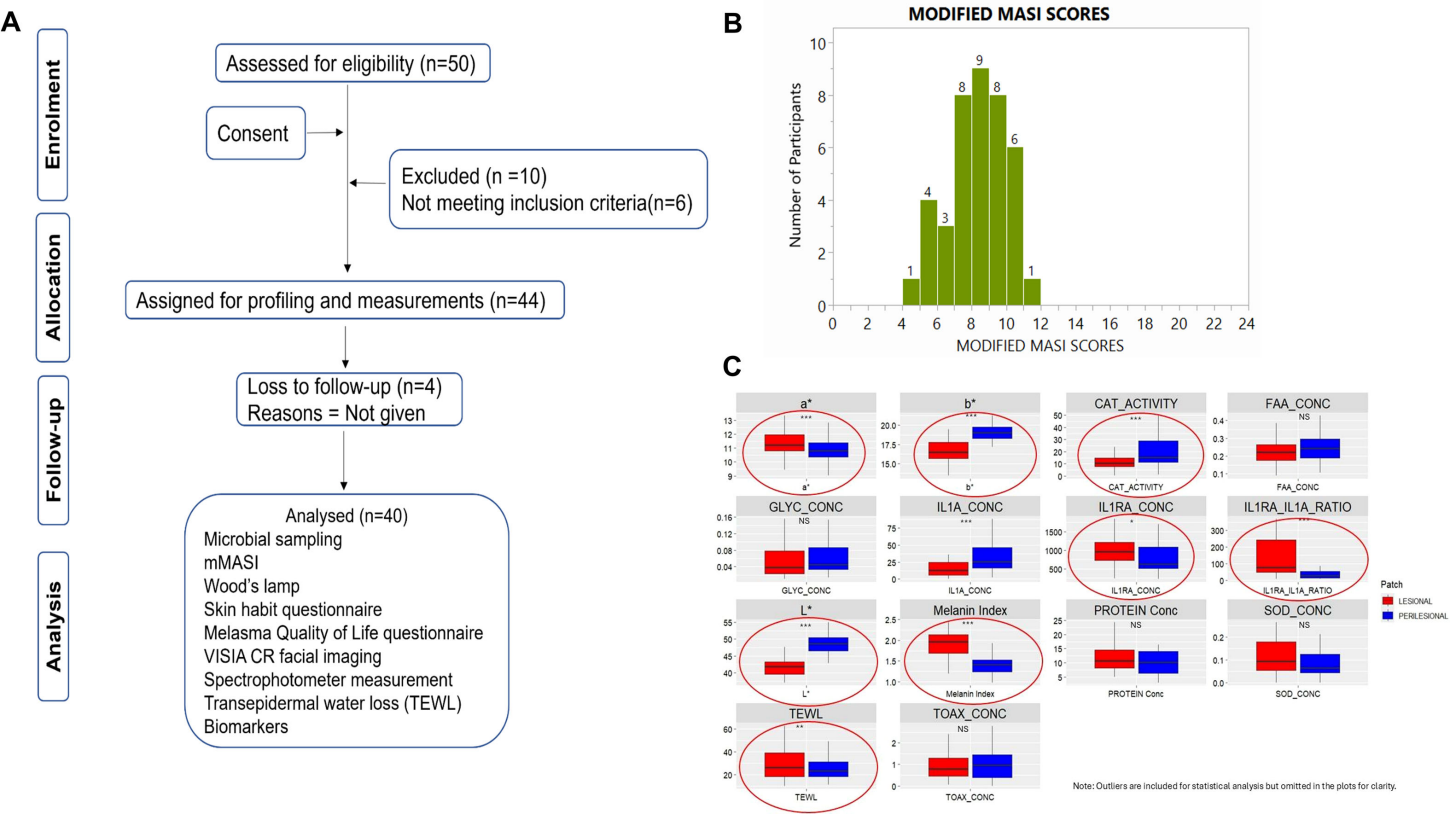
Clinical evaluation of melasma. **(A)** Schematic of the cohort development elaborates the process of clinical diagnosis, cohort development and clinical evaluation; **(B)** mMASI distribution plot; **(C)** Clinical parameters measured at lesions and peri-lesions. Parameters showing significant differences between lesion and peri-lesions are highlighted in red circles. The symbol * is pvalue <0.05, ** is <0.001 and *** is <0.0001.

In the study population (**Figure 1A**), melasma was clinically characterized and was seen to be uneven, blotchy mainly in the malar region (cheeks), forehead and chin, with severity assessed using mMASI with a mean value of 8.19 (range:4.80 - 11.2) (**Figure 1B**).

Interestingly, of the various clinical parameters and biomarkers that were sampled and analysed, several showed significant differences between lesion and peri-lesion areas (**Figure 1C**). Specifically, we measured skin parameters L*, a*, b* from a widely used colour model, CIELAB (Jeon et al., 2014) which is commonly used to quantify the colour of the skin and determine the level of pigmentation. The L* value represents the lightness or brightness of a colour and ranges from 0 (black) to 100 (white). The a* value represents the position along the red-green axis of the colour space, while the b* value assesses the yellowness or blueness of the skin. Positive a* values lean towards red and negative values lean towards green. Similarly, positive b* values lean towards yellowish undertones and negative values lean towards bluish undertones. Lesional areas showed a significant decrease in L* values as compared to peri-lesional melasma. This was accompanied by a significant increase in melanin index (MI), the measure of the skin melanin content based on spectral reflectance, in lesional compared to peri-lesional melasma. Together, these two measures confirmed the hyper-pigmentary condition, consistent with the clinical assessment. Additionally, melasma lesional areas also showed significantly higher a* and lower b* as compared to peri-lesional areas. Taken together, the instrumental assessment of melasma showed significant differences between lesional and peri-lesional areas.

A combinatorial approach employing biophysical, biochemical and biomarker-based measures was employed to evaluate barrier properties between lesional and peri-lesional melasma. Melasma lesions showed significantly higher TEWL levels compared to peri-lesional areas, accompanied by trends in higher total protein levels and lower free amino acid levels. Overall, evaluation of barrier properties based on measured parameters, indicated altered barrier properties associated with melasma.

Next, we measured biomarkers related to inflammatory status such as Interleukin-1α, Interleukin-1 receptor antagonist (IL1RA), and computed their ratios (IL1RA/IL1α) (**Figure 1C**). Interestingly, while we observed very significant increases in IL1α in non-lesion as compared to lesion areas, the levels of IL1RA were higher in lesions, resulting in very significant increase in the ratios of IL1RA/IL1α. Additionally, measurement of catalase activity as an indicator of redox regulation indicated significantly reduced levels between lesions and peri-lesions. However, the difference in SOD and total antioxidant activity was not significant. Overall, the data indicated perturbations in inflammatory status and compromised redox regulatory mechanisms in lesions as compared to peri-lesions.

**Figure 2:**
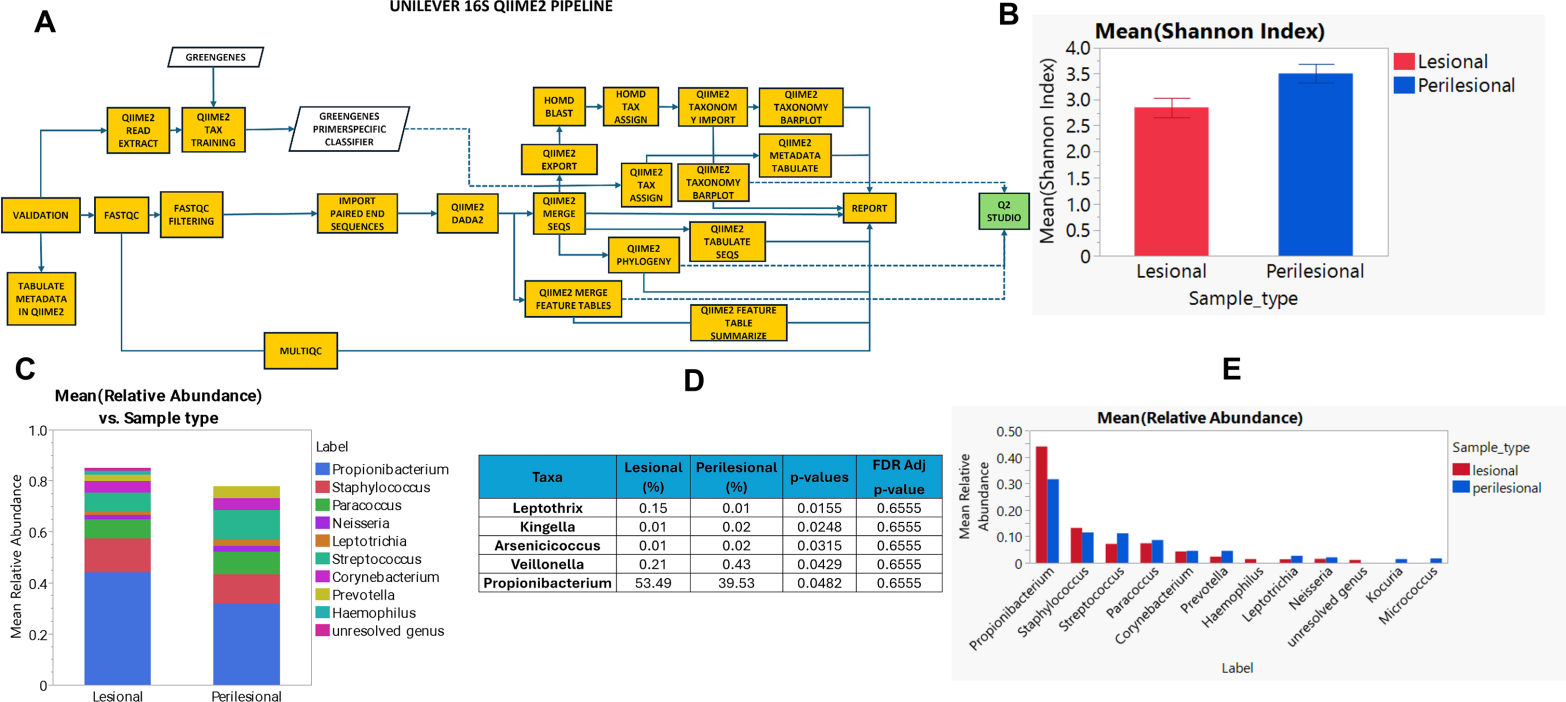
Microbiome profiling. **(A)** Schematic of the microbiome profiling through 16S & downstream analysis; **(B)** Alpha diversity assessed using the Shannon Index; **(C)** Visual differences in Mean Relative Abundances at genus level between lesion and peri-lesion sites; **(D)** Top five perturbed genera between lesion and peri-lesion sites with a p-value <0.05; **(E)** Mean relative abundance of the top ten perturbed genera across lesion and peri-lesion sites.

Microbiome profiling through 16S rRNA sequencing (**Figure 2A**) indicated the presence of expected bacterial genera in the skin such as the *Propionibacterium/Cutibacterium*, *Staphylococcus* in both lesional and peri-lesional samples. Overall, 377 genera were identified when all operational taxonomic units (OTUs) were agglomerated at the Genus level, with 344 being common and 12 being unique to lesional and 21 unique to peri-lesional areas. In an analysis to identify the core microbiome (prevalence in ≥ 90% of samples), 5 OTUs were found to be common to both lesion and peri-lesions, with none unique to lesion and 4 OTUs unique to peri-lesion. The 4 unique OTUs were identified as *Kocuria, Micrococcus, Pseudomonas and Acinetobacter*. Interestingly, the Alpha diversity as assessed using the Shannon index indicated a statistically significant difference (P = 0.0129) (**Figure 2B**), with a decrease in diversity in the lesional sites. This indicated a reduction in the species richness and evenness in the lesions. On the other hand, beta diversity was found to be not significantly different, indicating that the microbial community composition was similar in both groups (data not shown). Since the samples are paired with a lesional and peri-lesional control sample in each individual, we analysed the microbiomes pair-wise and found significant differences in the Mean Relative Abundances of some microbes in each individual, as depicted in the stack plot for the top ten genera (**Figure 2C**). Differences with significant p-values at the genus level between lesional and peri-lesional sites were observed in five taxa (**Figure 2D**), with Propionibacterium/Cutibacterium (P = 0.0482), being high in abundance and showing an increase in the lesional sites. The remaining four organisms were much lower in abundance overall, *Leptothrix* (P = 0.0155) showing an increase in lesions, whereas *Kingella* (P = 0.0248), *Arsenicicoccus* (P = 0.0315) and *Veillonella* (P = 0.0429) showing a decrease. *Propionibacterium/Cutibacterium* was the most abundant organism in both lesional & peri-lesional sites (**Figure 2E**).

### Melasma lesional areas have a sparser network as compared to peri-lesional areas

**Figure 3:**
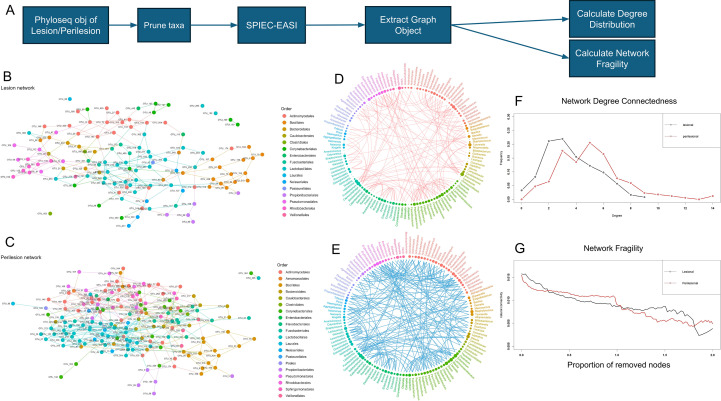
Microbiome community structure. **(A)** Schematic of SPIEC-EASI network generation; **(B-E)** Co-occurrence networks at OTU & Genus level; **(F)** Network degree connectedness; **(G)** Network fragility.

The lesional and peri-lesional areas were analysed using SPIEC-EASI package to generate networks so as to understand the underlying interactions in the microbial communities (**Figure 3A**). The co-occurrence networks showed subtle differences between both communities. A large number of connections were seen between the constituents to form a single large, connected network in both conditions with a few orphan nodes. The lesional areas had lesser number of interconnections and lesser nodes in comparison to the peri-lesional areas. This pattern continued to be seen even when different cutoff parameters were applied. The lesion network had 123 nodes with 220 edges, while the peri-lesion network had 170 nodes with 329 edges (**Figures 3B–E**). The network differences were subtle, with a few key nodes losing degree connectedness leading to sparser interactions rather than major replacement of key nodes as well a general reduction in number of degree connectedness across the entire network. A plot of degree connectedness of lesion vs peri-lesion illustrates this observation (**Figure 3F**). However, the lesion network was not more fragile than the peri-lesion as seen in the fragility analysis (**Figure 3G**). The lesion and peri-lesion network had *Streptococci*, *Micrococcus* and *Kocuria* as nodes with greater number of connections. While *Staphylococci* was far more connected in peri-lesion, it had lost most connections in the lesion. The lesion had an unresolved genus of the Lactobacillales order as the most connected node which is interestingly a much less important node in the peri-lesion. Among the highly connected nodes, *Lachnoanaerobaculum* and *Microbacterium* were the ones which was uniquely present in the peri-lesion network and absent in the lesion. The other more connected and unique nodes in the peri-lesion were *Fusobacterium* and *Leptotrichia.*

### Alterations in the lesional microbiome group into distinct subtypes

**Figure 4:**
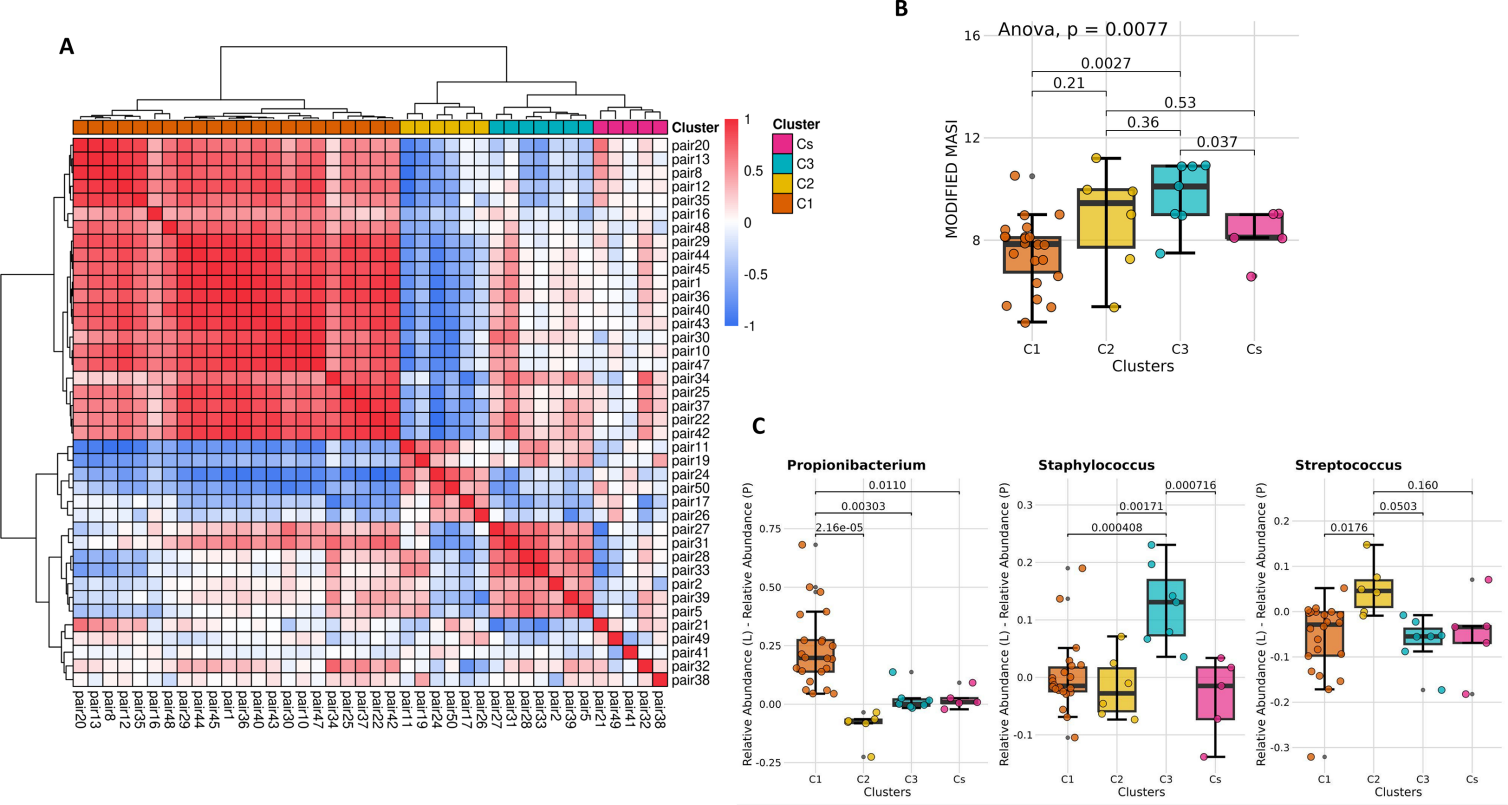
Pairwise correlation analysis. **(A)** Pairwise correlation across samples based on differences in the relative abundance of prevalent genera highlighting three clusters (C1, C2, and C3) and the presence of singletons (Cs; **(B)** Distribution of mMASI scores (ANOVA p-value: 0.0077) across the four groups; **(C)** The top enriched genera across the three clusters (Propionibacterium in C1, Streptococcus in C2 and Staphylococcus in C3) with FDR adjusted p-values of Tukey HSD tests.

Next, we tested if there were any subtypes among the 40 pairs in terms of their microbiome alterations in the lesional melasma. To achieve this, we carried out an agnostic clustering by taking those microbes that were present in majority of the samples (as described in the methods). Overall, 32 genera qualified for this analysis, which were used as an input into clustering. The clustering pattern indicated subtypes, since three clear clusters (C1, C2, and C3) and singletons or doublets grouped into a fourth cluster (Cs) were observed, of which two (C1 (n=22, within cluster mean R = 0.7556304) and C3 (n=7, within cluster mean R = 0.6470189)) were significantly distinct from each other (**Figure 4A**).

In comparison, a similar clustering with the same input series of the peri-lesional areas alone showed much more homogeneity (Average Pearson correlation index of 0.5193224 was seen as compared to a value of 0.1807556 seen for the lesion-peri-lesion pairs). This analysis indicated that the melasma lesions exhibited a significant amount of heterogeneity, which was evident despite the low sample size. Next, we tested if there was any pattern in the melasma severity scores among the subtypes and found that the subtype 1 had the least mMASI score (mean =7.52), whereas subtype 3 had the highest mMASI score (mean=9.76) (**Figure 4B**). We then analysed if there was any identifiable pattern of preponderances of genera in the lesions of these subtypes. We observe a statistically significant difference between the subtypes showing a higher abundance of *Propionibacterium/Cutibacterium* in lesions of subtype 1 (versus corresponding peri-lesions), whereas there was a higher abundance of Staphylococci in subtype 3. The subtype 2 had intermediate severity and was rich in *Streptococci* (**Figure 4C**). This seemed to suggest that *Staphylococci* could be associated with higher severity, whereas *Propionibacterium/Cutibacterium* could be associated with lower severity of melasma. We however note that the sample size is low, and a conclusive statement would require sampling a much larger size for the different subtypes.

We also analysed the measured clinical parameters versus all 32 genera (regardless of clusters) and observed several interesting positive and negative correlations. Notably, mMASI positively correlated (p-value=0.0017), while L* negatively correlated (p-value=0.017) with *Actinomyces* genera. Additionally, b* which was decreased in lesion versus peri-lesion areas, was observed to negatively correlate with *Rothia* genera (p-value=0.0414). The IL-1RA/IL-1α ratio which was found to be significantly increased in lesion as compared to peri-lesion melasma, negatively correlated with *Arthrobacter* (p-value=0.0447) and *Acinetobacter* (p-value=0.0185) genera. Catalase activity which was significantly lower in lesion as compared to peri-lesion melasma, positively correlated with organisms belonging to *Fusobacterium* (p-value=0.0158), *Gemella* (p-value=0.002) and *Haemophilus* (p-value=0.0469) genera. Lastly, protein content which was observed to be directionally higher in lesion as compared to peri-lesion areas positively correlated (p-value =0.0432) with *Enterobacter*, while negatively correlating with organisms belonging to *Gemella* genera (p-value=0.0195). (**Table 1**). Overall, our data indicated that differences in clinical parameters between lesion and peri-lesion melasma correlated (positively or negatively) with one or more organisms belonging to the top 32 genera.

**Table 1:** Correlation between clinical parameters and top 32 genera.

Clinical Parameter	Organism	Correlation	r-value	p-value
MODIFIED_MASI	*Actinomyces*	Positive	0.4805	0.0017
L*	*Actinomyces*	Negative	-0.3753	0.017
b*	*Rothia*	Negative	-0.324	0.0414
MELANIN Index	*Leptotrichia*	Positive	0.3613	0.022
IL1RA_CONC	*Arthrobacter*	Negative	-0.3874	0.0135
IL1RA_IL1A_RATIO	*Arthrobacter*	Negative	-0.3192	0.0447
	*Acinetobacter*	Negative	-0.3708	0.0185
SOD_CONC	*Actinomyces*	Negative	-0.332	0.0363
	*Alloprevotella*	Negative	-0.3421	0.0307
	*Bacillus*	Positive	0.6259	<0.0001
	*Paracoccus*	Positive	0.3766	0.0166
	*Prevotella*	Negative	-0.4761	0.0019
	*Veillonella*	Negative	-0.3156	0.0473
TOAX_CONC	*Brachybacterium*	Positive	0.4279	0.0059
	*Brevundimonas*	Positive	0.5987	<0.0001
	*Sphingomonas*	Positive	0.6875	<0.0001
	*Staphylococcus*	Negative	-0.3188	0.045
PROTEIN_CONC	*Enterobacter*	Positive	0.3214	0.0432
	*Gemella*	Negative	-0.3678	0.0195
CAT_ACTIVITY	*Fusobacterium*	Positive	0.3214	0.0158
	*Gemella*	Positive	0.475	0.002
	*Haemophilus*	Positive	0.3161	0.0469
GLYC_CONC	*Staphylococcus*	Negative	-0.3927	0.0122

## Discussion

Skin is home to a multitude of microbial communities belonging to 18 phyla, the four dominant ones being *Actinobacteria* (51.8%), *Firmicutes* (24.4%), *Proteobacteria* (16.5%) and *Bacteroidetes* (6.3%) (Grice et al, 2009). Though rich in bacterial density and second only to the gut (Cundell, 2018), our understanding of the skin microbiome is still in its infancy. However, advances in the field in the last decade have clearly indicated the role of the microbiome in skin health and diverse skin pathologies. Our goal in this work was to characterize the microbiome dysbiosis in lesions of melasma, a cutaneous hyperpigmentary disorder with poorly understood pathology. To control for variability in the normal skin flora at different times and among different people, we compared the microbiomes in lesions versus their adjacent peri-lesions and identified the dysbiosis patterns. We indeed observed a significant reduction in skin biodiversity in lesions. A decrease in skin biodiversity has been previously reported for other cutaneous pathologies, such as AD (Paller et al, 2019) and psoriasis (Tett et al, 2017). The most over-abundant genus in the lesions was *Propionibacterium/Cutibacterium.* While its high prevalence in sebaceous rich areas of the face is well documented (Wilson, 2005), its increase in melasma lesions is interesting, given that *C. acnes* was shown to influence pigmentation via Toll-like receptors (TLRs) (Koike et al, 2018), suggesting a linkage with pigmentation via an innate immune pathway. This is consistent with an earlier study (Hu et al, 2022) which reported that *Propionibacterium/Cutibacterium* was not only significantly enriched in melasma lesions but also correlated with the activation degree of melanocytes in melasma-involved areas. We speculate that the increased abundance of *Propionibacterium/Cutibacterium* may influence microbial diversity on the one hand by rendering the microenvironment less favourable to other microorganisms, and drive pigmentation on the other hand, through activation of the immune system. Organisms such as *Leptothrix, Kingella, Arsenicicoccus* and *Veillonella* were also significantly different between the lesion and peri-lesion with *Leptothrix* increasing in melasma lesions while *Kingella, Arsenicicoccus* and *Veillonella* decreasing in abundance in the lesional areas. Our analysis clearly pointed to a significant decrease in reduction in the species richness in melasma lesions. To study the impact it had, we constructed co-occurrence networks in lesions and peri-lesions and carried out a comparative analysis. Co-occurrence network analysis are a theoretical construct of the community structure of the microbiome, given the composition. While they have a limitation of typically representing statistical correlations and not true ecological interactions, they have been used to derive insights into the robustness and interconnectedness of the microbiomes through metrics such as mean degree, edge number, node number and network robustness (Saheb Kashaf et al, 2021; Bouslimani et al, 2015). Our analysis showed that the networks between the lesion and peri-lesion were visibly different and the number of edges as well the degree connectedness were changed, with the lesion network being sparser. A healthy microbiome often has dense, redundant interactions that buffer against change. Sparsity on the other hand, is indicative of community disruption, possibly due to inflammation in the lesions. Inflammation brings about several changes in the skin such as pH, lipid composition, oxygen levels, and immune environment, leading to reshaping the architecture of the microbial community by selecting for fast-growing, stress-resistant taxa (Hajar and Zhou, 2023; Baker et al, 2023). *Streptococci, Micrococcus* and *Kocuria* had greater number of connections in both lesion and peri-lesion areas in contrast to *Staphylococci*, a key skin commensal. Interestingly, both *Kocuria* and *Micrococcus* were reported as one of the taxa that had high correlation with high pigmentation spots (Zanchetta et al, 2022). Several S*taphylococci* are critical commensals in the skin microbiome, and it is likely that the reduction in their abundance might adversely affect the delicate balance between the microbiome and skin functions. *Lachnoanaerobaculum* and *Microbacterium* were uniquely present in the peri-lesion and absent in the lesion network, and highly connected. Both these microbes have been reported in skin, however their direct effects on pigmentation or inflammation are currently unknown. Altered co-occurrence networks have been previously reported in skin conditions eg: acne (Kim et al, 2021) and vitiligo (Gangu et al, 2016), as well as in response to external insults such as pollution (Wang et al, 2021). Overall, these studies indicate a role for the microbiomes and possibly their community network structures in pigmentation phenotypes whether temporary changes as in tanning (Willmott et al, 2023), or more sustained as in melasma (Hu et al., 2022) or vitiligo (Gangu et al, 2016).

Multiple studies have reported changes in inflammatory markers in melasma lesions (Kang et al, 2002; Kwon et al, 2016; Liu et al, 2023). Consistent with that, our results indicate an inflammatory status in melasma lesions as seen in the levels of the proinflammatory cytokine IL-1α and its receptor antagonist, IL-1RA. A significant decrease in IL-1α in melasma lesions accompanied by increase in IL1RA levels resulted in a very significant increase in the ratios of IL1RA/IL-1α similar to trends reported in chronically sun-exposed skin (Hirao et al, 1996). While such a change is reflective of skin responses from chronically sun-exposed skin, it is tempting to speculate that a heightened ratio may be linked to microbial dysbiosis.

Measurement of oxidative stress-related markers such as catalase activity, SOD and total antioxidant activity in our study showed a very significant decrease in catalase activity, with no significant change in either SOD or Total antioxidant activity in melasma lesions. While a similar reduction in serum catalase was reported (Sarkar et al, 2020), to the best of our knowledge, this is the first report of reduced catalase activity on skin of melasma subjects. On the other hand, reported increase in serum SOD activity and reduced plasma glutathione in melasma is contrary to our findings (Kuthial et al, 2019; Wiraguna et al, 2020). The observed discrepancies may be due to differences in regulatory mechanisms governing skin antioxidant systems in the local versus systemic milieu. While oxidative stress can be induced by both external and internal stressors, notably, UVR, pollution, sleep deprivation, etc., (Esposito et al., 2021) in the context of melasma, the exact causes leading to oxidative stress and the relative contribution of local versus systemic perturbations that activate antioxidant defence mechanisms have not been deciphered. It is possible that the reduced anti-oxidant activity in melasma lesions is possibly linked to the observed microbial dysbiosis.

We also observed changes in barrier properties and associated markers in the lesions. Given the intricate relation between host and microbiome, barrier perturbations and its likely impact on the microbiome are not surprising. Multiple studies have reported barrier defects in melasma lesions (Lee et al, 2012; Kang et al, 2011; Xu et al, 2021). Trends for increase in Transepidermal water loss in melasma lesions upon barrier perturbation with tapestripping in our study are in line with previously reported studies. However, reduced stratum corneum cohesivity and NMFs such as Free Fatty Acids (FFAs) provides a previously unreported dimension to altered barrier function in melasma. Increased Profilaggrin expression in malar melasma subjects was reported by (Castanedo-Cazares et al, 2023). Since degradation of Filaggrin protein contributes to the cytosolic pool of NMFs (Rinnov et al., 2023), observed increase in their study could be due to recovery mechanisms deployed to restore barrier function via the Filaggrin-NMF axis.

Further analysis identified three distinct microbial clusters through an agnostic clustering approach, indicating that the microbial population was significantly more heterogeneous in lesions. Concomitant variations in melasma severity scores across the three clusters (with C1 having the least, and C3 having the highest scores), raised the intriguing possibility that microbiome heterogeneity in the lesions could underlie clinical heterogeneity. The diverse clinical features exhibited by patients were previously shown to be accompanied by varying degree of histological features, highlighting complex etiopathogenesis, that could in turn influence disease maintenance, exacerbation, recurrence and/or treatment outcomes (Lee, 2015; Artzi et al, 2021). Our study points to the microbiome as yet another potential contributor to the highly heterogeneous pathology in melasma. Further, the higher abundance of *Staphylococci* in C3 (having highest melasma severity scores) as compared to higher abundance of *Propionibacterium/Cutibacterium*) in C1 (having lowest melasma severity scores) was unexpected yet interesting. Prominent members of this genera such as *S. epidermidis* have a well- established role in skin health; it was shown to impair skin barrier and elicit skin inflammation in mice when grown at high density via a cysteine protease enzyme EcpA. Interestingly, in atopic dermatitis, abundance of *S. epidermidis* and expression of EcpA was shown to correlate with disease severity (Cau et al, 2021). Likewise, *S. epidermidis* was shown to switch from a commensal to a pathogen and activate skin inflammation under excess growth conditions (Williams et al, 2023). In the correlational analysis, while significant correlations were seen between several organisms with pigmentation (mMASI), none have been reported to be able to directly influence pigmentation in any previous studies. Our observations of correlations (both positive and negative) between clinical parameters, notably catalase activity, inflammatory markers, protein content and organisms belonging to the top 32 genera supports the intimate relation between host and microbiome that may underlie complex melasma pathogenesis. Taken together, our novel findings indicate that microbiome is heterogeneous in melasma, and this could in turn modulate skin functions to varying extents ultimately influencing clinical heterogeneity.

A limitation of our study is the small sample size especially in view of the heterogeneity in the severity of the condition, making it difficult to apply statistical methods for correcting for multiple testing. Nevertheless, our results clearly indicate the microbiome alterations in the lesions which could have a possible physiological link to alterations in skin pigmentation. The current methodologies of microbiome analysis provide correlations of individual taxa or genera with the condition of interest, but it would be interesting to aim to establish causality in the future.

## Conclusions

Our study of microbiomes in melasma lesions as compared to their matched peri-lesion controls clearly indicates microbial dysbiosis in the lesions with concomitant changes in barrier, redox and inflammatory status in the host. Further, our findings that the microbiomes in the lesions group into distinct clusters which exhibit variation in the clinical parameters such as the mMASI scores, raises the possibility that the microbiome may influence clinical heterogeneity. Overall, teasing apart the intricate links between host and microbiome may open up simple and effective intervention strategies to tackle this recalcitrant disorder.

## Data Availability

The datasets presented in this study can be found in online repositories. The names of the repository/repositories and accession number(s) can be found below: https://www.ncbi.nlm.nih.gov/, PRJNA1138279.
