# Supplementary material for: Microbial dysbiosis in melasma through community profiling
Source: Front Microbiomes. 2025 Dec 22;4:1505565. doi: 10.3389/frmbi.2025.1505565 (PMC12993618; doi:10.3389/frmbi.2025.1505565)
Supplement: Supplementary file 1 [file DataSheet1.docx]

**Supplementary Information 1**

**Inclusion Criteria**

1. Subjects willing to provide written consent.
2. Subjects of age between 25 to 45 years inclusive.
3. Subjects having skin type III-IV
4. Subjects with 50 to 69% (as per the area scores of the mMASI scale) coverage area of melasma on both sides of the cheeks
5. Subjects having moderate to severe facial melasma pigmentation (as per the Melasma severity scale) which has been stable for 6 months (epidermal/dermal/mixed type, to be assessed using Wood’s lamp).

**Exclusion Criteria**

1. Subjects who have undergone any treatment for melasma /chronic melasma like laser therapy, bleaching agents, or any other skin lightening treatment (topical or oral) in past 6 months prior to sample collection
2. Subjects using oral contraceptives or on hormone replacement therapy.
3. Suffering from autoimmune diseases like thyroid autoimmunity or diabetes mellitus.
4. Use of topical antibiotics or topical steroids past 4 weeks.
5. Use of the following drugs at least 6 months prior to sampling.
   1. Systemic antibiotics (Oral, intravenous, intramuscular).
   2. Oral corticosteroids
   3. Cytokines or immunosuppressive agents
6. Subjects who are pregnant, lactating or nursing.
7. Any underlying uncontrolled medical illness including diabetes and hypertension, liver disease or history of alcoholism, HIV, hepatitis or any other serious medical illness.
8. Subject in an exclusion period or participating in any other cosmetic or therapeutic study.
9. Intense sun exposure/ photo allergenicity/toxicity.
10. Subjects who have undergone facial cosmetic procedures including chemical peels, microderm abrasion, or laser treatment within 6 months.
