# Supplementary material for: Microbial dysbiosis in melasma through community profiling
Source: Front Microbiomes. 2025 Dec 22;4:1505565. doi: 10.3389/frmbi.2025.1505565 (PMC12993618; doi:10.3389/frmbi.2025.1505565)
Supplement: Supplementary file 2 [file DataSheet2.docx]

**Supplementary Information 2**

Representative images of lesions from cheek areas of subjects depicting the heterogeneity of melasma severity, taken using VISIA CR imaging system.

**
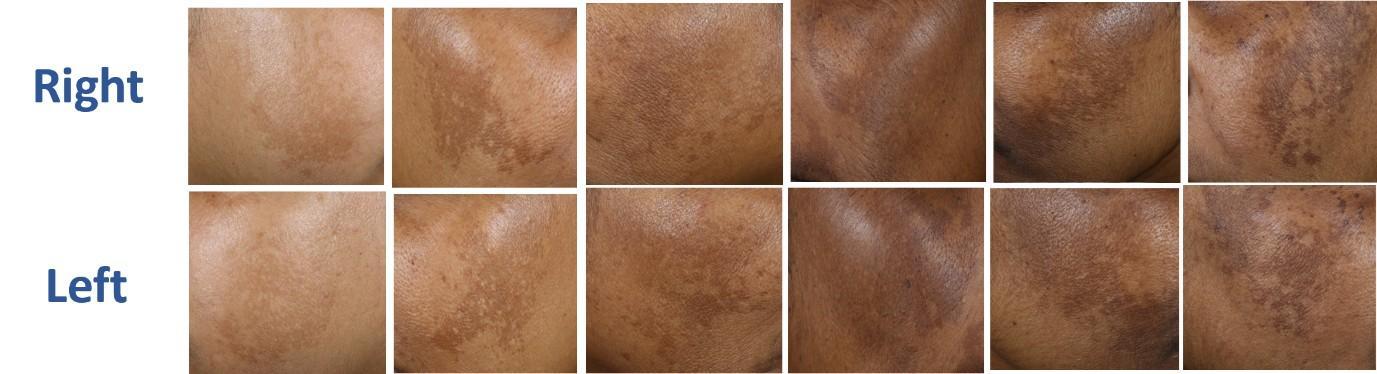
**
