# Supplementary material for: Microbial dysbiosis in melasma through community profiling
Source: Front Microbiomes. 2025 Dec 22;4:1505565. doi: 10.3389/frmbi.2025.1505565 (PMC12993618; doi:10.3389/frmbi.2025.1505565)
Supplement: Supplementary file 3 [file DataSheet3.docx]

**Supplementary Information 3**

**Study visit 1 (enrolment and screening visit) – Day (-8)**

The subjects were screened for study inclusion-exclusion criteria and quailed subjects were enrolled after obtaining informed consent. Sufficient number of subjects were enrolled in the study to complete the study with a total of 40 adult female subjects. Depth of melasma (dermal/mixed/epidermal) was identified using Wood’s lamp and the severity of melasma was scored using the mMASI and melasma severity scales.

Subjects were provided Dove soap bar for use for at least seven days prior to visit 2. They were asked to wash their face with soap and water at home at least 24 hrs before visit 2 and record the time of wash in the subject diary.

**Study visit 2 (Day 1)**

Subjects were allowed to acclimatize in a temperature and humidity-controlled temperature of 20-22°C and relative humidity of 40% - 60%)clean and dry room for at least 2 hrs on the day of sampling. The sampling room and materials used were pre-sterilized for 30 min with UV just before sampling. Subjects' hair was secured with a headband to avoid any contamination while sampling. Technicians and all other study personnel present in the sampling room covered their face and hair with a face mask and hair net respectively while sampling to avoid any external contamination. Sampling room-specific footwear was used. Fresh sterile gloves were used after each sampling. Four sites on the face, two on each cheek Lesion and peri-lesional were identified for sampling. With a freshly opened sterile swab dipped in sampling buffer the entire lesional / peri-lesional was swabbed gently for 30 sec (~25to 30 strokes). Individual swabs- i.e., swabs collected from Right lesional, left lesional, right peri-lesional and left peri-lesional were severed using sterile scissors in its respective labelled sterile tubes containing 600 microliters lysis buffer (component of DNA extraction kit). Tubes were stored in ice packs until further processing. Post microbiome sampling, subjects washed their face with water. Skin color was measured at lesional and peri-lesional sites using spectrophotometer (CM 2600d).

**Study visit 3 (Day 2)**

Subjects were acclimatized in an air-conditioned room (with dehumidifier facility), maintained with a temperature of 20-22°C and relative humidity of 40% - 60% for about 15 to 30 mins. Skin barrier was measured at lesional and peri-lesional sites using Tewameter. The same test sites were selected for tape-stripping using D-squame tapes. Barrier was also measured after 5^th^ and 10^th^ tape collection and 5 hours post tape stripping process. Subjects were instructed not to wash or wet their face until completion of visit 4. Visit 4 should be scheduled 24 hours after visit 3.

**Study visit 4 (Day 3)**

Subjects were acclimatized for 15-20 minutes under controlled temperature and humidity- controlled room (20-22°C temperature and RH-40-60%). Barrier was measured 24 hours±2 hours post initial barrier measurement at visit 3.
