# Supplementary material for: Microbial dysbiosis in melasma through community profiling
Source: Front Microbiomes. 2025 Dec 22;4:1505565. doi: 10.3389/frmbi.2025.1505565 (PMC12993618; doi:10.3389/frmbi.2025.1505565)
Supplement: Supplementary file 4 [file DataSheet4.docx]

**Supplementary Information 4**

The 16srDNA data analysis is represented below


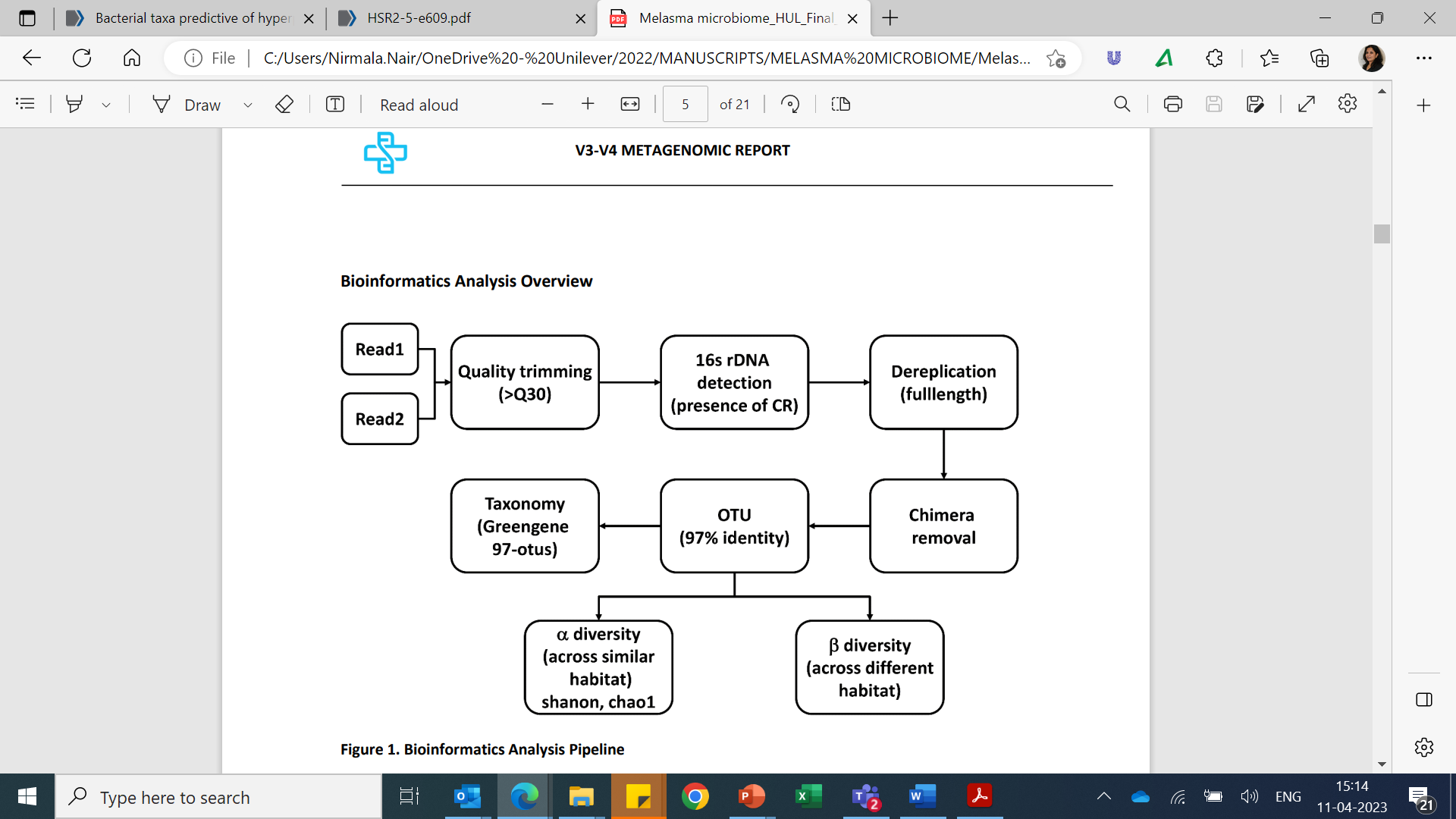


**Bioinformatics Analysis Overview**
