# Supplementary material for: Microbial dysbiosis in melasma through community profiling
Source: Front Microbiomes. 2025 Dec 22;4:1505565. doi: 10.3389/frmbi.2025.1505565 (PMC12993618; doi:10.3389/frmbi.2025.1505565)
Supplement: Supplementary file 5 [file DataSheet5.docx]

**Supplementary Information 5**

**Biomarker analysis through tape stripping**

1. **Chemicals, reagents, and assay kits**

Metmyoglobin, 2, 2’-azino-di-[3-ethylbenzthiazoline sulphonate] (ABTS) and 4-amino-3- hydrazino-5-mercapto-1, 2, 4-triazole (Purpald), Xanthine oxidase, Superoxide dismutase, free glycerol reagent (F6428), Ophthaladehyde (OPA), N-benzoyl- L-arginine ethyl ester (BAEE) was purchased from Sigma Aldrich chemicals (St Louis, USA). The IL-1α and IL-1ra in the SC were quantified using specific enzyme-linked immunosorbent assay kit (ELISA; R&D systems) according to the manufacturer’s instructions. The Micro BCA protein assay kit was obtained from Pierce (USA). All other chemicals were of analytical grade. The D-squame discs (D100) were obtained from CuDerm (Dallas, USA).

Total protein and biomarker detection kits were purchased from:

**Total protein**: Micro BCA kit - ThermoFisher, cat # PI23235

**TOAX**: Cayman chemicals, cat # 709001

**Catalase**: Cayman chemicals, cat # 707002

**SOD:** Cayman chemicals, cat # 706002

**Glycerol assay kit**: Cayman chemicals, cat # 10010755

**Free amino acids**: Using OPA reagents (mentioned above).

1. **Measurement of catalase activity**

This method is based on the peroxidative function of CAT, wherein a lower alcohol like methanol acts as a hydrogen donor, resulting in the formation of formaldehyde (Johansson and Borg 1998). CAT CH3OH + H2O2 HCHO + 2 H2O The detection of CAT activity on tape strippings from SC was modified from Hellemans et al (2003). Briefly, D-squames were placed in 1.5 ml eppendorf tubes in a solution of 5.9 M methanol and 4.2 mM hydrogen peroxide in 100 mM phosphate buffer pH 7.0. The tubes were vortexed well and left at room temperature for 20 minutes in sonicating water bath. After incubation the tape strippings were removed and the remaining solution was used for the quantification of formaldehyde. Formaldehyde was quantified spectrophotometrically at 550 nm after reaction with 4-amino-3-hydrazino-5-mercapto-1, 2, 4-triazole (Purpald), followed by complete oxidation with potassium periodate at high pH (pH 14). CAT activity was defined as μ moles of formaldehyde formed / min. Specific CAT activity was obtained after normalizing to total protein content of the D-squame.

1. **Measurement of total antioxidant (TOAX) activity**

The method is based on the ability of antioxidants in the samples to inhibit the oxidation of ABTS (2, 2’-azino-di-[3-ethylbenzthiazoline sulphonate]) by Metmyoglobin (a peroxidase). The amount of ABTS produced can be monitored by reading the absorbance at 750 or 405 nm. Under the reaction conditions the antioxidants in the sample cause suppression of the absorbance at 750nm or 405nm to the degree which is proportional to their concentration. The capacity of antioxidants in the sample to prevent ABTS oxidation is compared with that of trolox, a water-soluble tocopherol analogue and is quantified as mM trolox equivalents. Small molecule Antioxidants ABTS + Metmyoglobin +H2O2 Decrease in absorbance at 750/405nm This method does not separate the aqueous and lipid-soluble antioxidants, and therefore it is the combined antioxidant activities of all the non-enzymic antioxidants in the SC including vitamins, proteins, lipids, glutathione, uric acid etc.

1. **Measurement of superoxide dismutase (SOD)**

In this method xanthine oxidase in the presence of oxygen and hypoxanthine produces superoxide anion (O2 .-). A tetrazolium salt utilizes the liberated O2. - to form a yellow-coloured formazan dye, which exhibits absorbance at 450nm. The SOD present in the tape strip inhibits the oxidation of the tetrazolium salt by scavenging the superoxide anions and there by suppresses the absorbance at 450nm to the degree which is proportional to its concentration in the tape strip. SOD activity of samples was calculated using the equation obtained from the linear regression of the standard curve substituting the linearised rate (LR) for each sample. Specific activity of SOD was obtained after normalizing to total protein content of the D-squame.

1. **Detection of glycerol**

Each D-squame strip was subjected to 20 min sonication in 500μl of 1x phosphate buffered saline (PBS), followed by centrifugation at high speed for 5min. The supernatant collected was used for glycerol estimation. This assay measures glycerol by a coupled enzymatic reaction system. The glycerol present in the sample undergoes phosphorylation by glycerol kinase (GK) to produce glycerol-1 phosphate (G-1-P) and adenosine-5’-diphosphate (ADP). The G-1-P gets oxidized by glycerol phosphate oxidase (GPO) producing dihydroxy acetone phosphate (DAP) and hydrogen peroxide. Peroxidase (POD) catalyses the redox–coupled reaction of H2O2 with 4-aminoantipyridine (4-AP) and N-ethyl-N-(3-sulfopropyl)-m-anisidine (ESPA) producing a purple-coloured product with an absorbance maximum at 540nm.

1. **Detection of free fatty acids (FAA)**

Each D-squame strip was treated with 500μl of 0.01N HCl and incubated in room temperature for 2 hrs with constant shaking. The FAA contained in the extracted solvent was aspirated and transferred in to similarly labelled eppendorfs and stored at 4°C until analysis. FAAs were measured using O-phthaladehyde (OPA) from the acid hydrolysed extract. Briefly 100μl of OPA reagent was added to 100μl of acid extract in a 96-well plate. After 5 min the fluorescent intensity was measured with excitation at 340nm and emission at 465nm. L-Trp was used as an amino acid standard.

1. **Detection of IL-1α and IL-1RA**

The extraction of IL-1α from individual D-squame strips were done using 500ul of 1x phosphate buffered saline (PBS), followed by probe sonication for 10sec. The extracted interleukin was stored at 4°C until assay. The quantification of IL-1α was done using commercial ELISA kits according to the manufacture’s instruction. Specific IL-1α and IL-1RA activity were obtained after normalizing to total protein content of the D-squame. Protein content of the samples was quantified using BCA kit. The ratio of IL1α /IL-1RA were calculated using the respective values.

1. **Detection of total protein content**

The total protein content on the D-squame strippings was quantified using the BCA assay kit. Briefly, protein extraction from D-Squame was done in 1x phosphate buffered saline (PBS) by sonication in a water bath for 10min, followed by centrifugation at high speed for 5min. Supernatant collected was used for protein estimation. BCA method combines the well-known reduction of Cu^+2^ to Cu^+1^ by protein present in the sample in an alkaline medium (the biuret reaction) with the highly sensitive and selective colorimetric detection of the cuprous cation (Cu+1) using a unique reagent containing bicinchoninic acid. The purple-coloured reaction product of this assay is formed by the chelation of two molecules of BCA with one cuprous ion. This water-soluble complex exhibits a strong absorbance at 562 nm and the intensity of the colour is directly proportional to the concentration of protein in the sample.
